# Supplementary material for: Cellular and Humoral Response After Induction of Protection and After Finishing Hymenoptera Venom Immunotherapy
Source: Biomolecules. 2024 Nov 24;14(12):1494. doi: 10.3390/biom14121494 (PMC11673861; doi:10.3390/biom14121494)

Supplementary Material

Supplementary Table S1. Characteristics of patients.

Patient number	Age (years)	Sex	Grade of initial allergic reaction according to Muller scale	Double sensitization (honeybee and wasp venom)	Adverse systemic reactions on VIT
**Successful VIT**					
VIT_1	52	M	III	No	Itching of palms
VIT_2	27	M	II	Yes	Itching of scalp and groin
VIT_3	59	F	IV	No	No
VIT_4	27	M	III	Yes	No
VIT_5	44	M	III	Yes	No
VIT_6	53	M	IV	Yes	No
VIT_7	52	M	III	No	No
VIT_8	47	M	II	Yes	No
VIT_9	54	M	III	Yes	No
VIT_10	49	M	IV	Yes	No
VIT_11	51	M	IV	Yes	No
VIT_12	23	M	III	Yes	No

VIT_13	42	F	II	Yes	No
VIT_14	48	M	III	Yes	No
VIT_15	63	M	IV	Yes	No
VIT_16	41	F	IV	No	No
VIT_17	47	M	III	No	No
VIT_18	38	M	III	Yes	No
VIT_19	52	M	IV	Yes	No
VIT_20	57	M	II	No	No
**Treatment failure**					
VIT_21	49	M	IV	No	Yes
VIT_22	59	M	IV	No	Erythema of face
VIT_23	43	M	III	No	Itching of hands and legs
VIT_24	59	M	III	No	Erythema of face and palms
VIT_25	26	M	IV	Yes	Erythema and itching of palms, sleepiness

Supplementary Figure S1. Area under curve of basophils CD63 response of patients with successful venom immunotherapy.

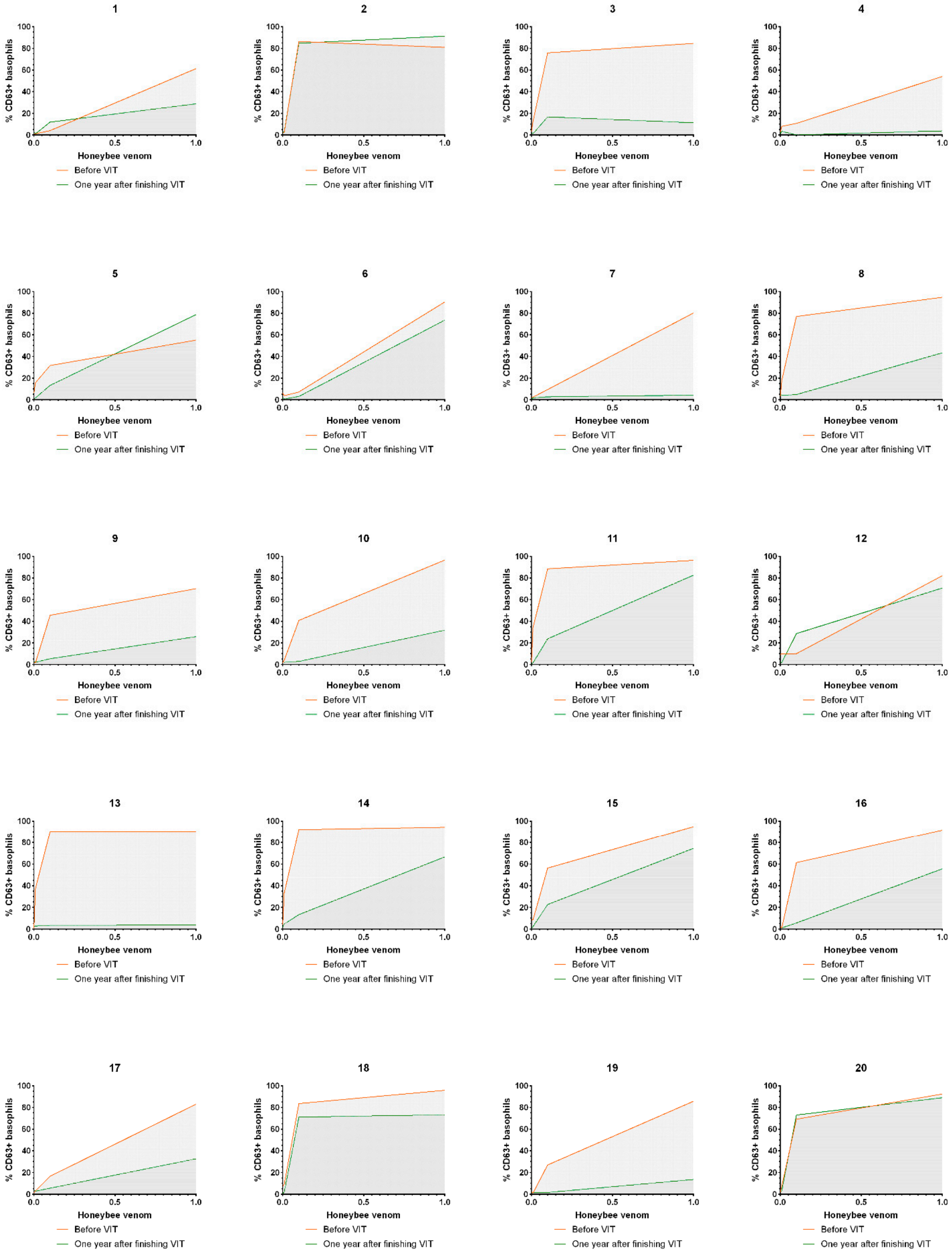

Supplementary Figure S2. Area under curve of basophils CD63 response of patients with treatment failure.

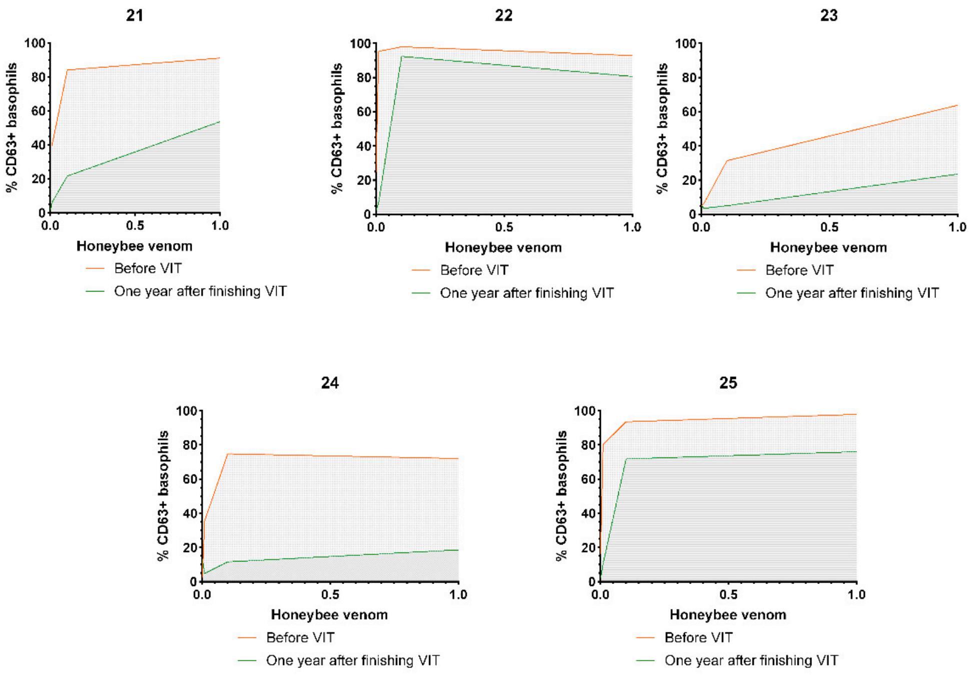

Supplementary Figure S3. AUC calculated from honeybee venom stimulation concentrations of 1 μg/mL, 0.1 μg/mL, 0.01 μg/mL, and 0.001 μg/mL. (A) Comparison before and after venom immunotherapy. (B) Percentage of change of AUC after VIT.

VIT, venom immunotherapy; AUC; area under curve; **** statistical significance p-value ≤ 0.0001, AUC; area under curve

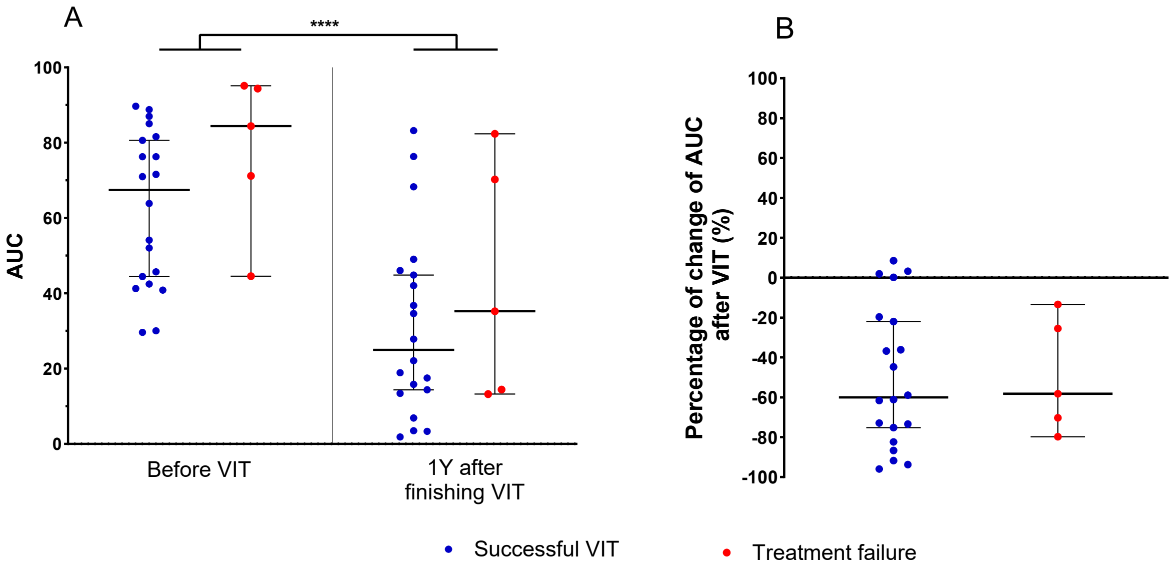

Supplement: Supplementary file 1 [file biomolecules-14-01494-s001.zip › biomolecules-3297406-supplementary.pdf]
